# Supplementary material for: The essential elements for the noncovalent association of two DNA ends during NHEJ synapsis
Source: Nat Commun. 2019 Aug 9;10:3588. doi: 10.1038/s41467-019-11507-z (PMC6688983; doi:10.1038/s41467-019-11507-z)
Supplement: Supplementary file 1 — Supplementary Information [file 41467_2019_11507_MOESM1_ESM.pdf]

Supplementary Information

The Essential Elements for the Noncovalent Association of Two DNA Ends During NHEJ Synapsis

Zhao et al.

Supplementary Figures

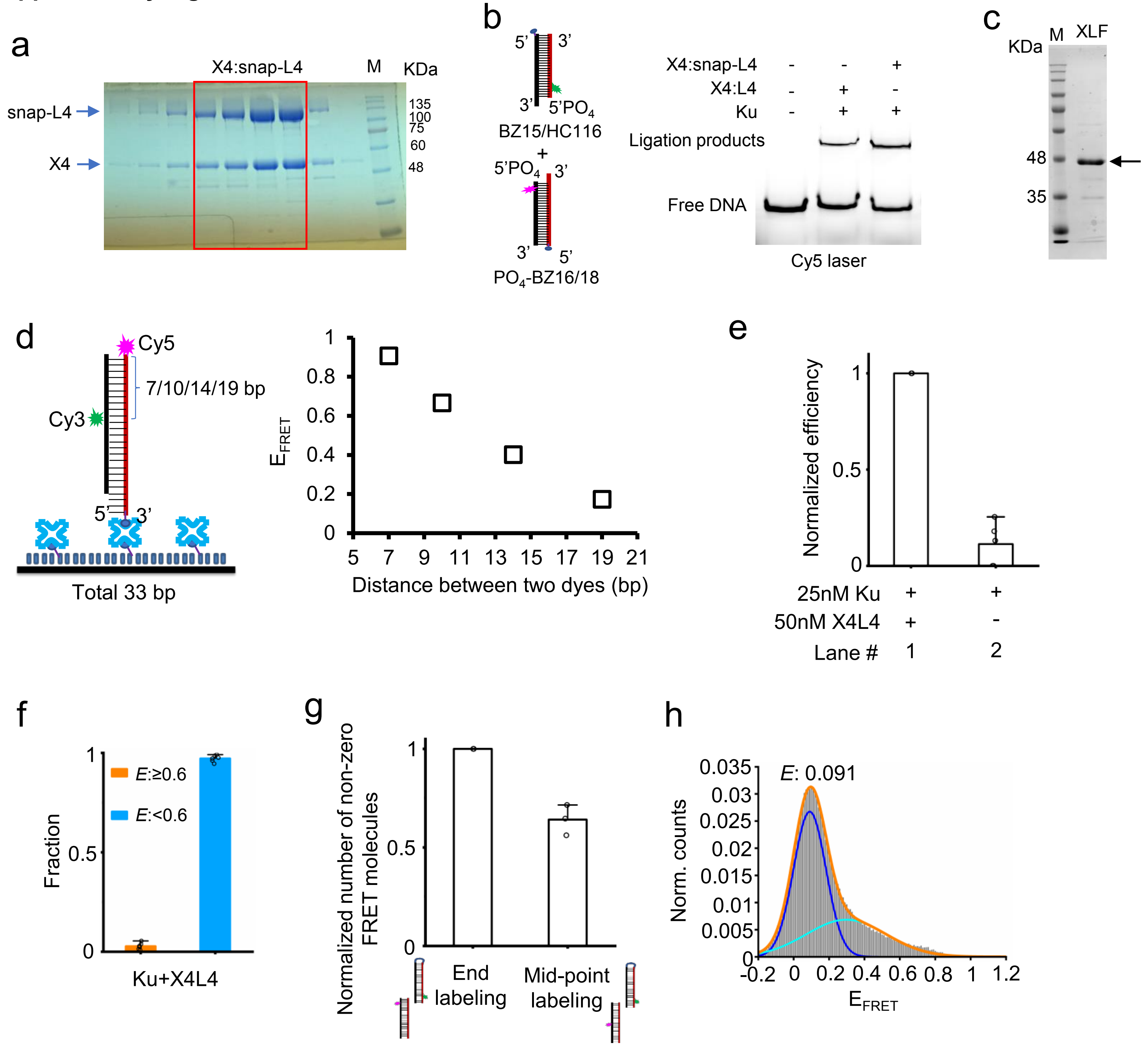

Supplementary Figure 1. Ku and X4L4 can mediate efficient synapsis.

a, Purified human X4:snap-L4 protein. The fractions marked by the red box were combined and used.

b, Purified human X4:snap-L4 exhibits equivalent ligation activity as X4L4 without snap-tag. BZ15 was synthesized with a PO_4 at the 5' end.

c, Purified human XLF protein.

d, Distance dependent E_{FRET} measurement using our system. The distance between two dyes are 7, 10, 14, and 19 bp.

e, Normalized synapsis efficiency mediated by different NHEJ factors - 25 nM Ku, 50 nM X4L4. Data are represented as mean ± SD of at least two independent replicates.

f, Fractions of different synaptic complexes formed by 25 nM Ku, 50 nM X4L4. Data is represented as mean ± SD of six replicates.

g, Normalized number of detected non-zero FRET molecules for DNA probes with acceptor dyes located at different positions (end labeling: Cy5 is 4 bp from the end; mid-point labeling: Cy5 is 40 bp from the end). 25 nM Ku and 50 nM X4L4 were used in the reactions. Data are represented as mean ± SD of three independent replicates.

h, Histogram of E_{FRET} values of all synapsis events from the middle labeling group mediated by 25 nM Ku and 50 nM X4L4. The *E* value shown was obtained by a Gaussian fit of the highest peak. N=202 molecules.

Source data are provided as Source Data1 and Source Data2 files.

a

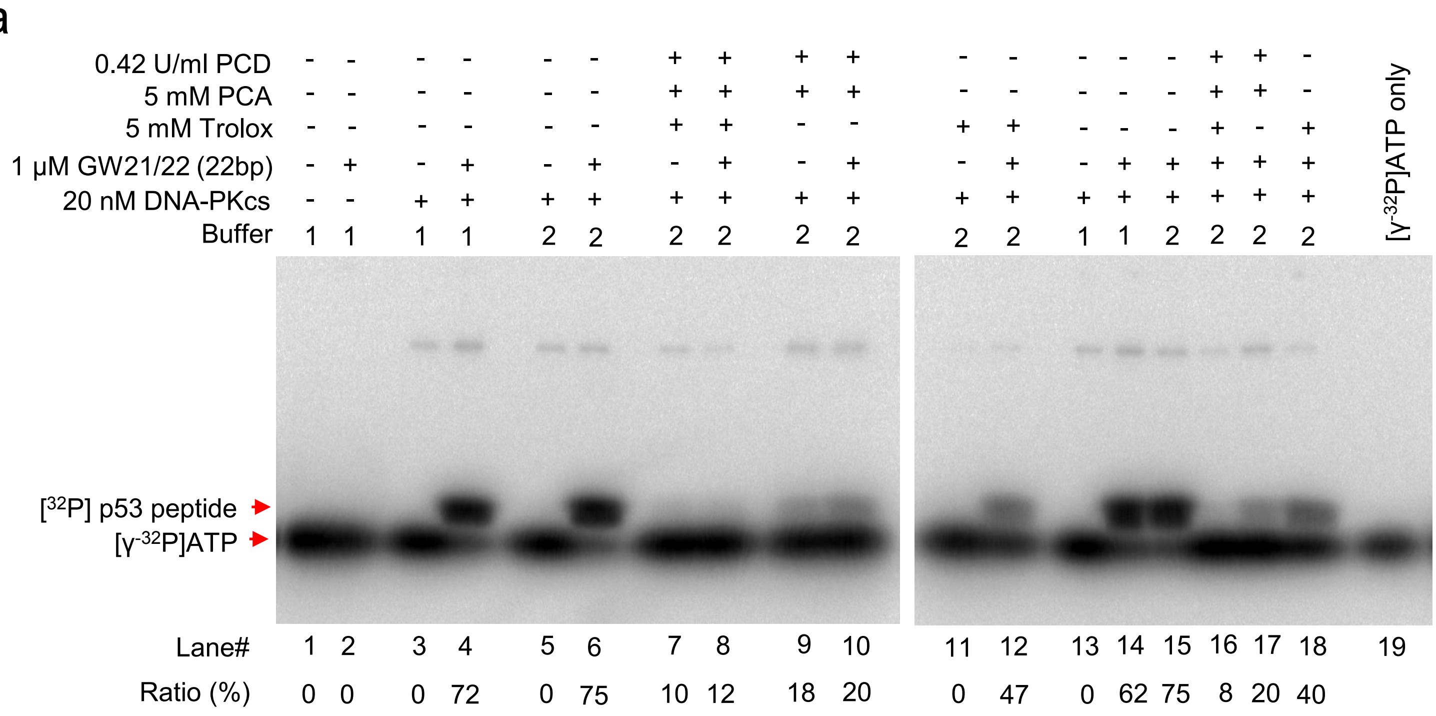

b

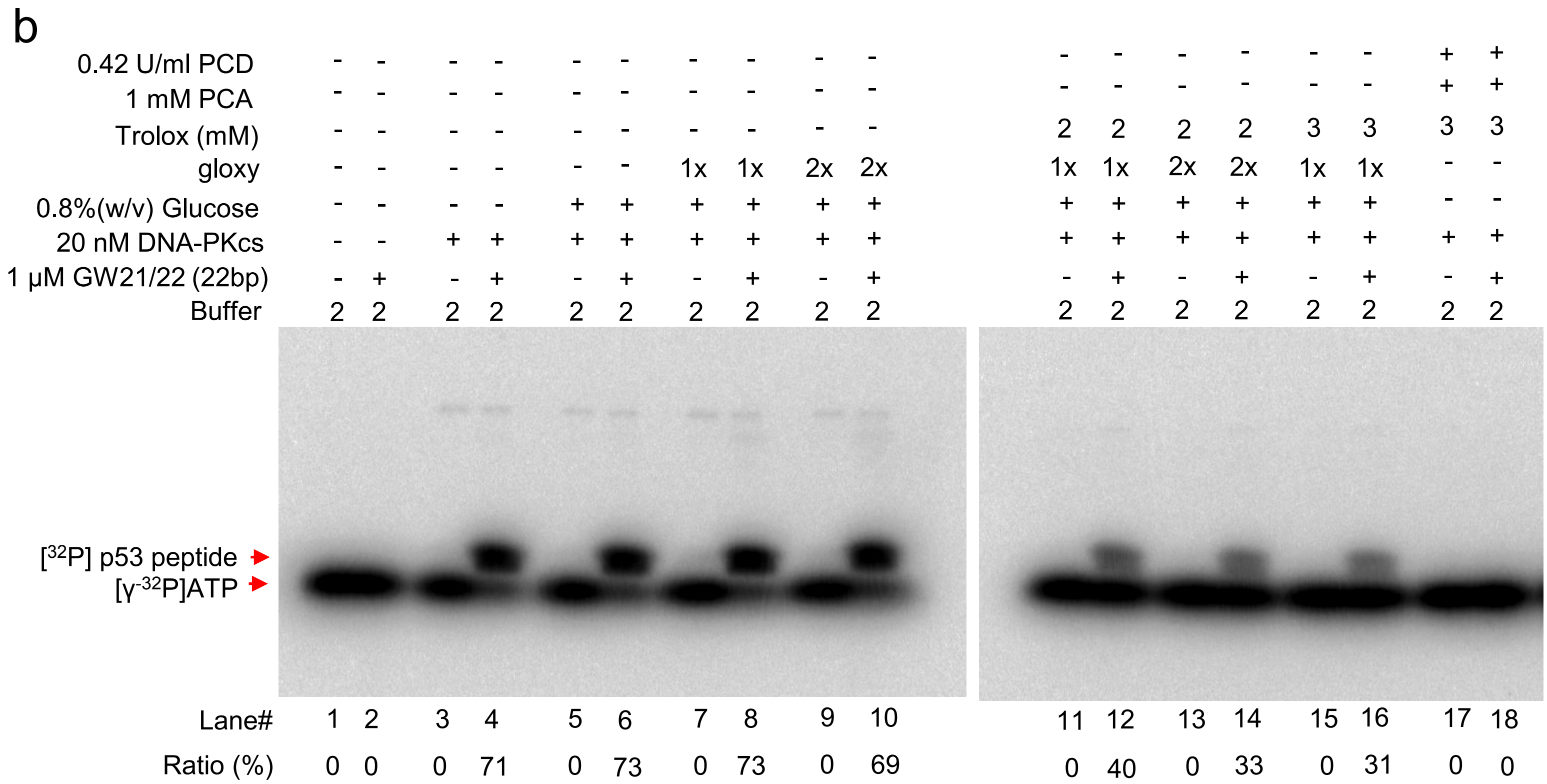

Supplementary Figure 2. Effect of different reagents on DNA-PKcs kinase activity.

PCA + PCD oxygen scavenger system severely reduces the kinase activity of DNA-PKcs (**a**). Glucose + Gloxy oxygen scavenger system can retain the kinase activity of DNA-PKcs (**b**). Buffer 1 consists of 25 mM Tris-HCl pH 7.5, 10 mM $MgCl_2$, 10 mM DTT, 5% Sucrose, and 80 nM [γ-^{32}P] ATP. Buffer 2 is the same as smFRET imaging buffer except the ATP. Buffer 2 consists of 20 mM Tris-acetate pH 7.5, 75 mM KCl, 10 mM $MgCl_2$, 3% glycerol, 1 mg/ml BSA, 2 mM DTT, 0.25% Tween 20, and 80 nM [γ-^{32}P] ATP. 1x gloxy consists of 165 U/ml glucose oxidase, and 2170 U/ml catalase.
Source data are provided as a Source Data2 file.

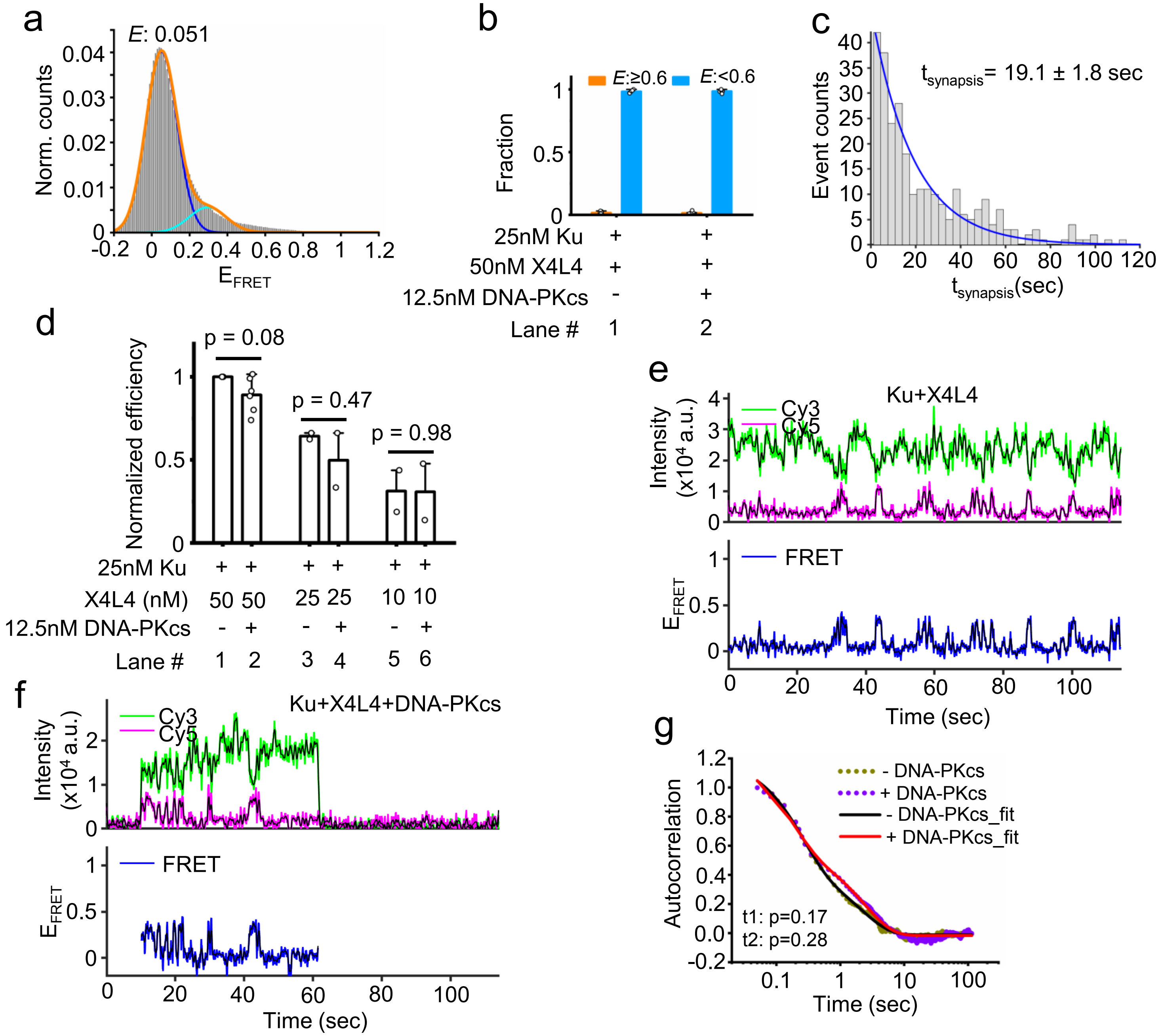

Supplementary Figure 3. Synapsis mediated by Ku, X4L4, and DNA-PKcs in the glucose plus gloxy oxygen scavenger system.

a, Histogram of E_{FRET} values of all synapsis events mediated by 25 nM Ku and 50 nM X4L4 in the glucose plus gloxy oxygen scavenger system. The *E* value shown on the graph was obtained from a Gaussian fit of the highest peak. N = 1078 molecules.

b, Fractions of different synaptic complexes formed by 25 nM Ku, 50 nM X4L4, and 12.5 nM DNA-PKcs in the glucose plus gloxy oxygen scavenger system. Data is represented as mean ± SD of six replicates.

c, Histogram and corresponding exponential fit of total synapsis time mediated by Ku and X4L4 in the glucose plus gloxy oxygen scavenger system. Cy3 signal lifetime (including zero-FRET and detectable FRET portions) of the synaptic complex was used to calculate the total dwell time for each synapsis event and only the synapsis events (n = 277) with both start and end time points within the detection time window were included. Synapsis time shown on the graph was represented as mean ± S.D. of three independent repeats.

d, Normalized synapsis efficiency. Data are represented as mean ± SD of at least two independent replicates. The data shown on lane 1 and lane 2 are the same as shown on Fig. 2a. The t-test (unpaired, two-tailed) was applied for p value calculation.

e,**f**, One of the dynamic intensity traces (donor: green; acceptor: magenta) and corresponding E_{FRET} values (blue) of synapsis mediated by Ku and X4L4 (**d**) or by Ku, X4L4, and DNA-PKcs (**e**) in the glucose plus gloxy oxygen scavernger system . The black line represents the smoothed trace of corresponding donor, acceptor, or E_{FRET} trace.

g, Autocorrelation curves for the dynamic synaptic complexes and corresponding bi-exponential fit. The synaptic complex was formed by Ku and X4L4, and by Ku, X4L4, and DNA-PKcs, and the study used the glucose plus gloxy oxygen scavenger system in the solution. The t-test (unpaired, two-tailed) was applied for p value calculation.

Source data are provided as a Source Data1 file.

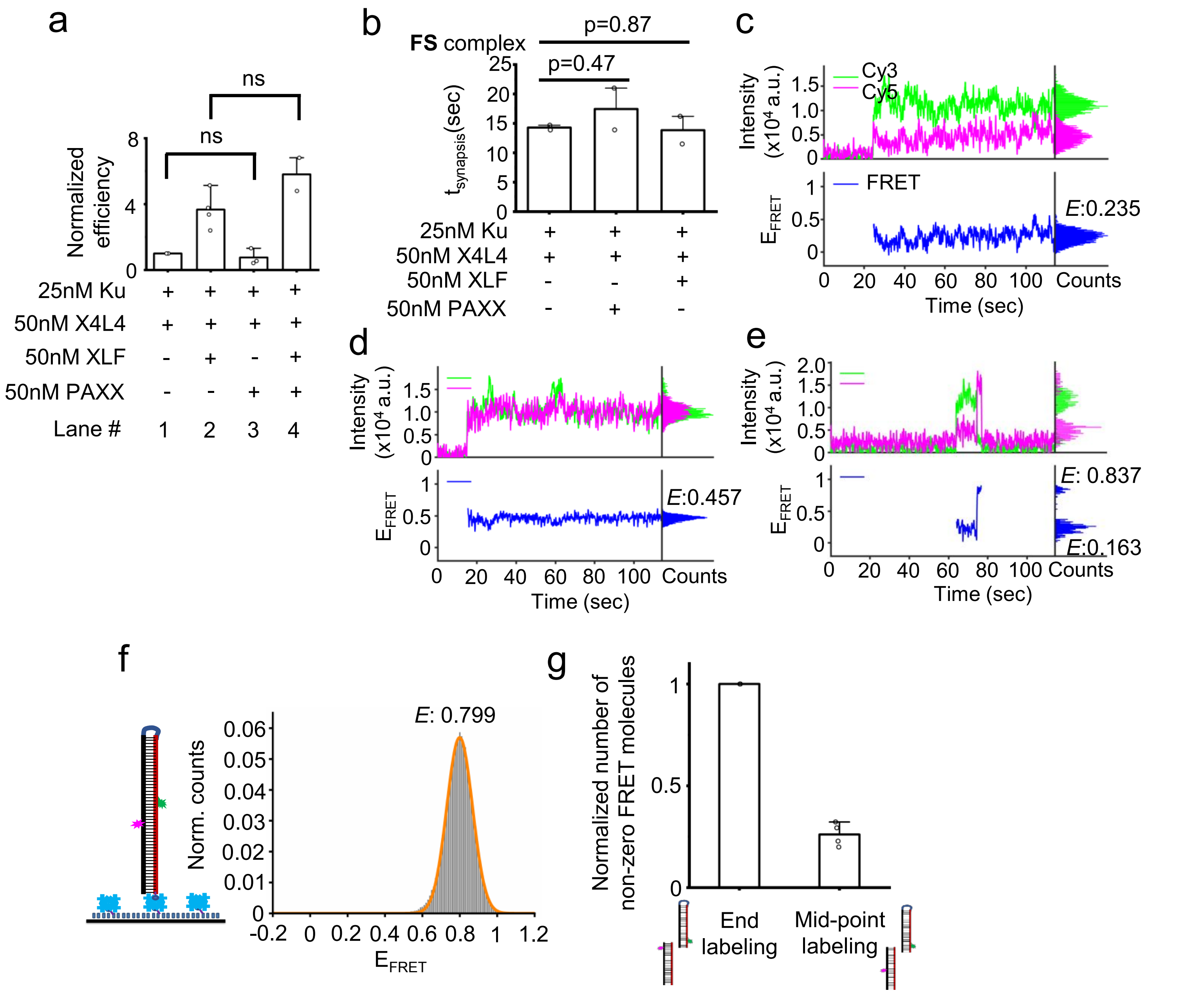

Supplementary Figure 4. Effect of XLF on synapsis mediated by Ku and X4L4.

a, Normalized synapsis efficiency. Data are represented as mean ± SD of at least two independent replicates. T-test (unpaired, two-tailed) was applied for p value calculation. ns represents not significant ($p>0.05$).

b, Dwell time of the low E_{FRET} **FS** complexes formed with or without XLF or PAXX present. Only the low E_{FRET} ($E < 0.6$) events with both start and end time points within the detection time window were included. Error bars represent SD of two replicates. T-test (unpaired, two-tailed) was applied for p value calculation.

c,**d**,**e**, Representative single molecule time traces of donor (green) intensity, acceptor (magenta) intensity and corresponding E_{FRET} values (blue) for synapsis mediated by 25 nM Ku, 50 nM X4L4 and 50 nM XLF. The right parts are histograms of donor intensity (green), acceptor intensity (magenta) and E_{FRET} values (blue) within synapsis period.

f, Histogram of E_{FRET} values from a pre-ligated control. The *E* value was obtained from a Gaussian fit of the peak. N=186 molecules.

g, Normalized number of detected non-zero FRET molecules for DNA probes with acceptor dyes located at different positions (end labeling: Cy5 is 4 bp from the end; mid-point labeling: Cy5 is 40 bp from the end). 25 nM Ku, 50 nM X4L4 and 50 nM XLF were used in the reactions. Data are represented as the mean ± SD of four independent replicates. Source data are provided as a Source Data1 file.

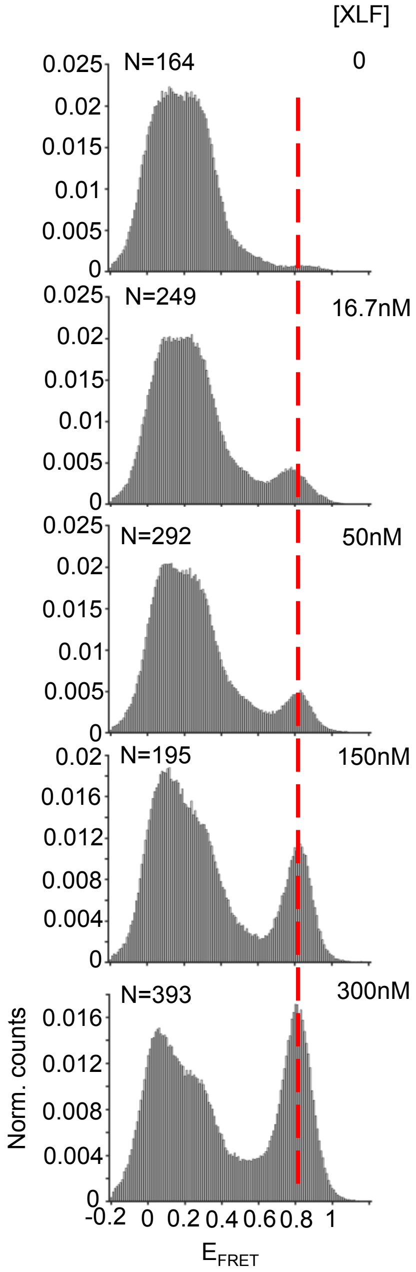

Supplementary Figure 5. XLF concentration dependence for the accumulation of the CS complex. Histograms of E_{FRET} values of all the synaptic complexes formed by 25 nM Ku, 50 nM X4L4 and different amounts of XLF. The synapsis data used here for E_{FRET} distribution graphs is the same batch as that used in Fig. 3**f** for fraction calculation. Source data are provided as a Source Data1 file.

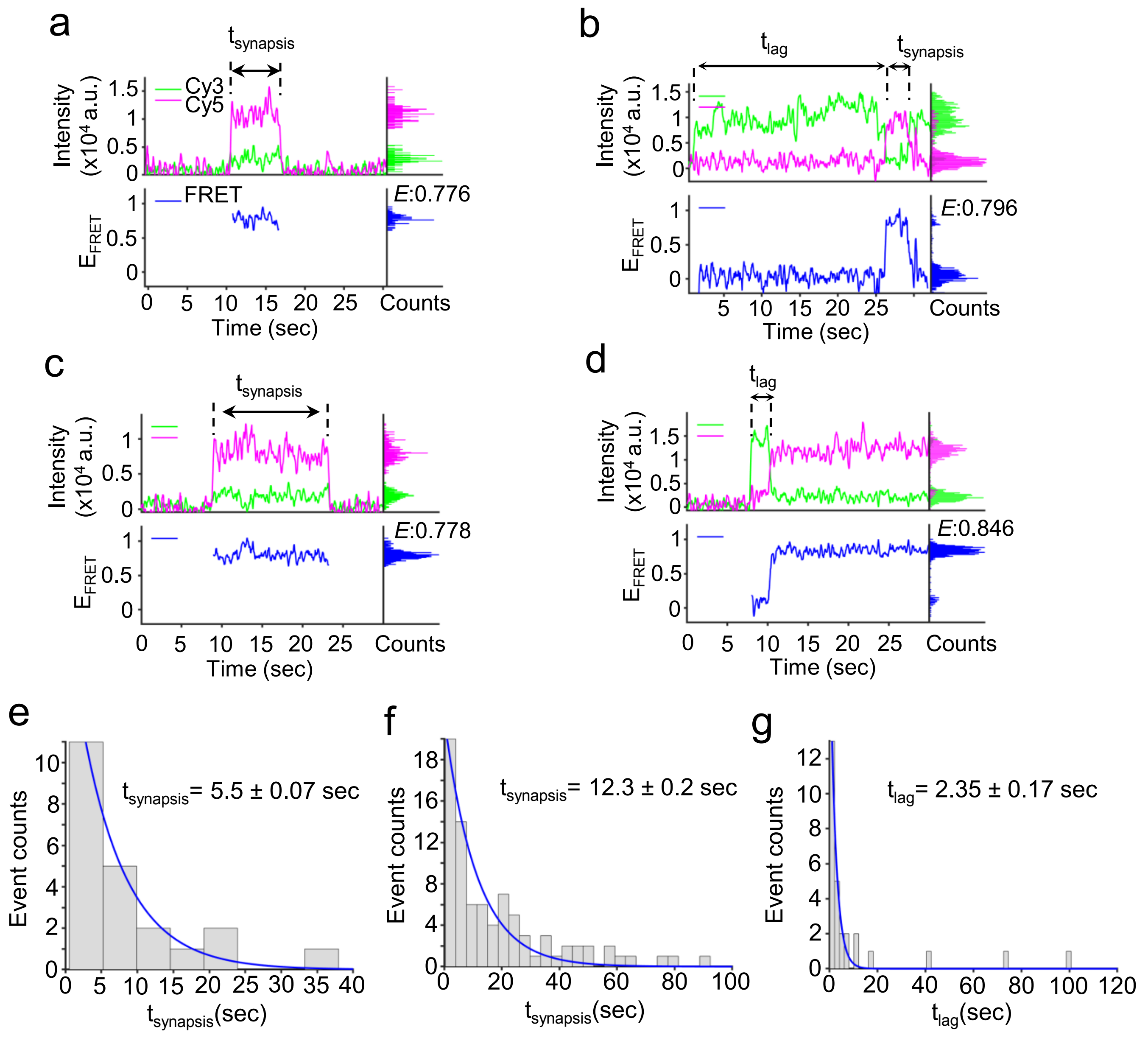

Supplementary Figure 6. PAXX increases end proximity of two dsDNA but with weak strength.

a,**b**,**c**,**d**, Representative single molecule time traces of donor (green) intensity, acceptor (magenta) intensity and corresponding E_{FRET} values (blue) for synapsis mediated by 25 nM Ku, 50 nM X4L4, and 50 nM PAXX (**a**,**b**), and by 25 nM Ku, 50 nM X4L4, 50 nM PAXX, and 50 nM XLF (**c**,**d**). The right parts are histograms of donor intensity (green), acceptor intensity (magenta) and E_{FRET} values (blue) within the synapsis period.

e,**f**, Histograms and corresponding exponential fits of synapsis times of the **CS** complex mediated by Ku, X4L4 and PAXX (**e**), and by Ku, X4L4, XLF and PAXX (**f**). Only the high E_{FRET} events (**CS** complex) with both start and end time points within the detection time window were included. n=22 traces for Ku, X4L4 and PAXX reaction; n=85 traces for Ku, X4L4, XLF and PAXX reaction. The fitted synapsis time is also summarized in Fig. 4**d**. Synapsis time shown on the graph was represented as mean ± S.D. of two replicates.

g, Histogram and corresponding exponential fit of the lag time between synapsis starting and transition to high E_{FRET} in the presence of XLF and PAXX. Only the events with a detectable transition from low E_{FRET} (E_{FRET} < 0.6) to high E_{FRET} (E_{FRET} ≥ 0.6) were selected. n = 28 traces. Error represents the SEM of the fit.

Source data are provided as a Source Data1 file.

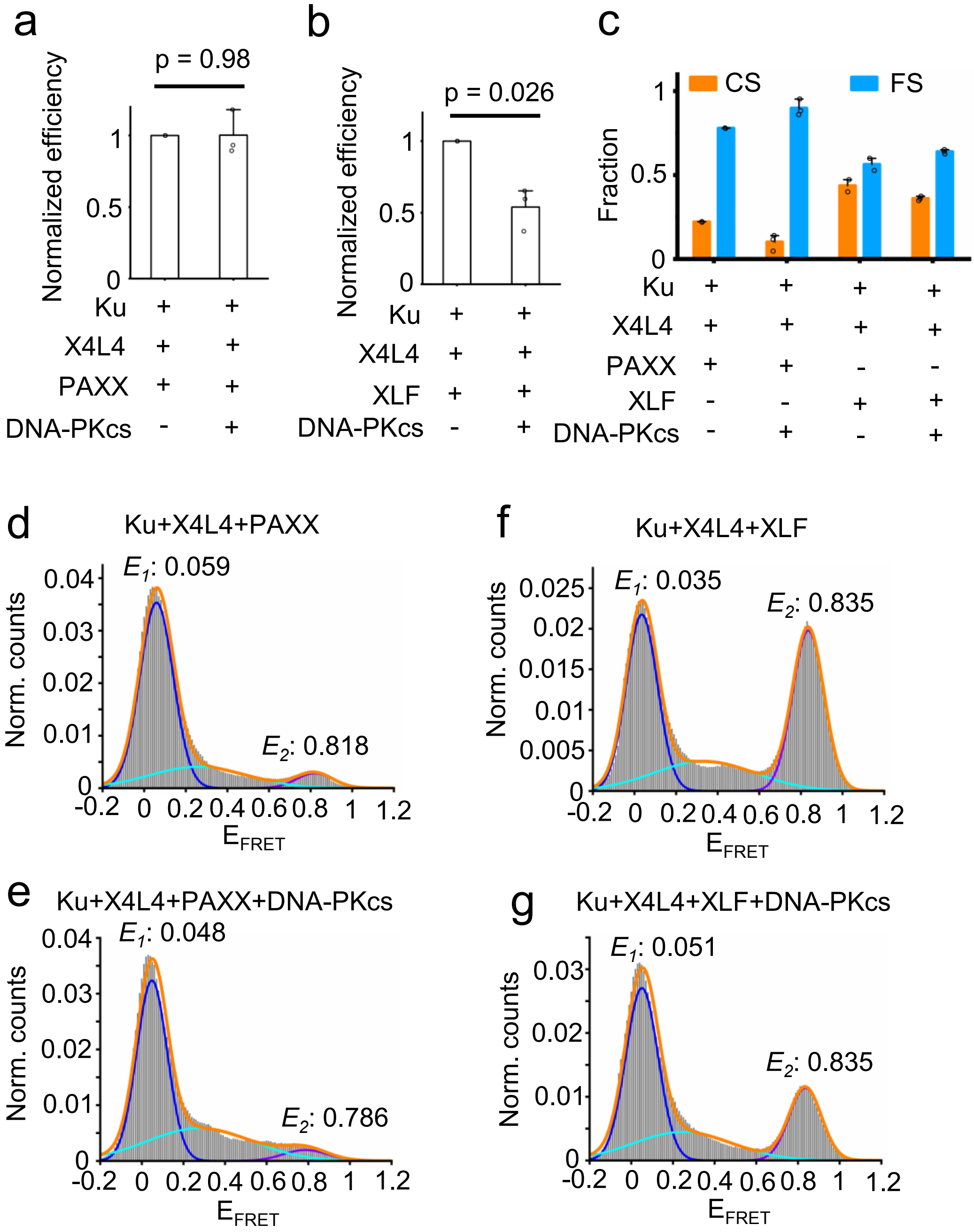

Supplementary Figure 7. Effect of DNA-PKcs on the synapsis mediated by Ku+X4L4+PAXX and Ku+X4L4+XLF in the glucose plus gloxy oxygen scavenger system.

a,**b**, Effect of DNA-PKcs on the total efficiency of synapsis (**FS**+**CS**) using the glucose plus gloxy oxygen scavenger system. Data are represented as mean ± SD of at least three independent replicates. T-test (unpaired, two-tailed) was applied for p values calculation. The proteins used are 25 nM Ku, 12.5 nM DNA-PKcs, 50 nM X4L4, 50 nM PAXX, and 50 nM XLF.

c, Fractions of different synaptic complexes formed by NHEJ proteins in the glucose plus gloxy oxygen scavenger system. The proteins used are 25 nM Ku, 12.5 nM DNA-PKcs, 50 nM X4L4, 50 nM PAXX, and 50 nM XLF. Data is represented as mean ± SD of at least two replicates.

d,**e**,**f**,**g**, Histograms of E_{FRET} values of all synaptic complexes mediated by Ku, X4L4 and PAXX (**d**), by Ku, DNA-PKcs, X4L4, and PAXX (**e**), by Ku, X4L4 and XLF (**f**), by Ku, DNA-PKcs, X4L4, and XLF (**g**) in the glucose plus gloxy oxygen scavenger system. The *E* value shown was obtained from a Gaussian fit of the highest peak of each kind of synaptic complex (**FS** or **CS**). Source data are provided as a Source Data1 file.

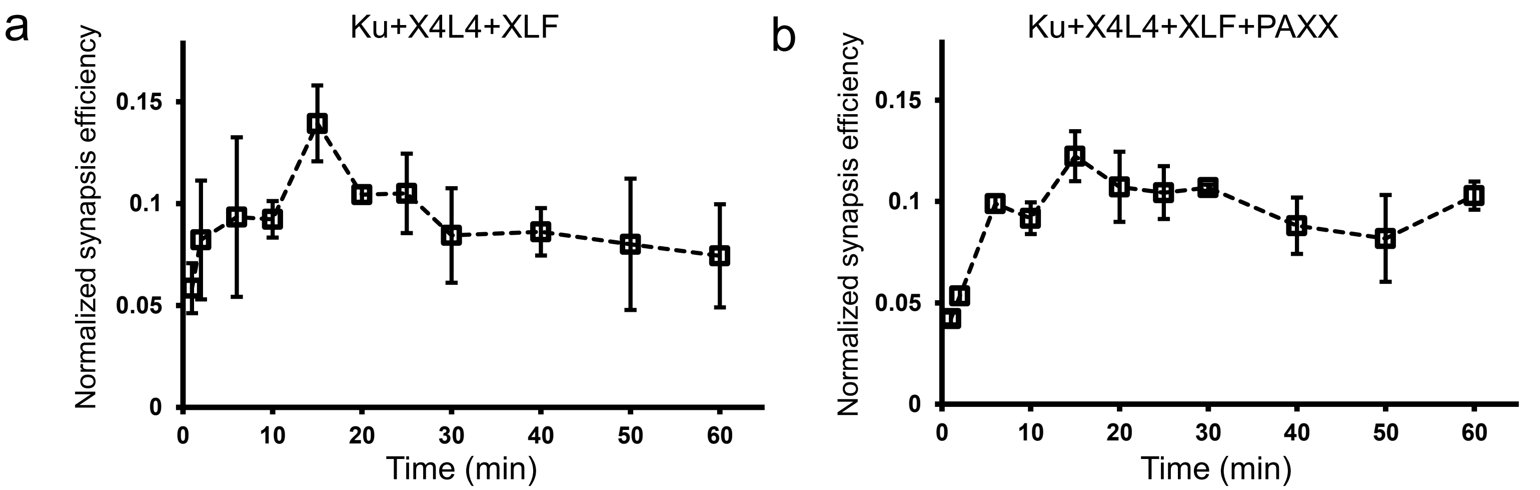

Supplementary Figure 8. Time-dependent synapsis efficiency.

a,**b**, Kinetics of total synaptic complex formation mediated by Ku, X4L4 and XLF (**a**) or by Ku, X4L4, XLF and PAXX (**b**). Data are represented as mean ± SD of two independent replicates.

Source data are provided as a Source Data1 file.

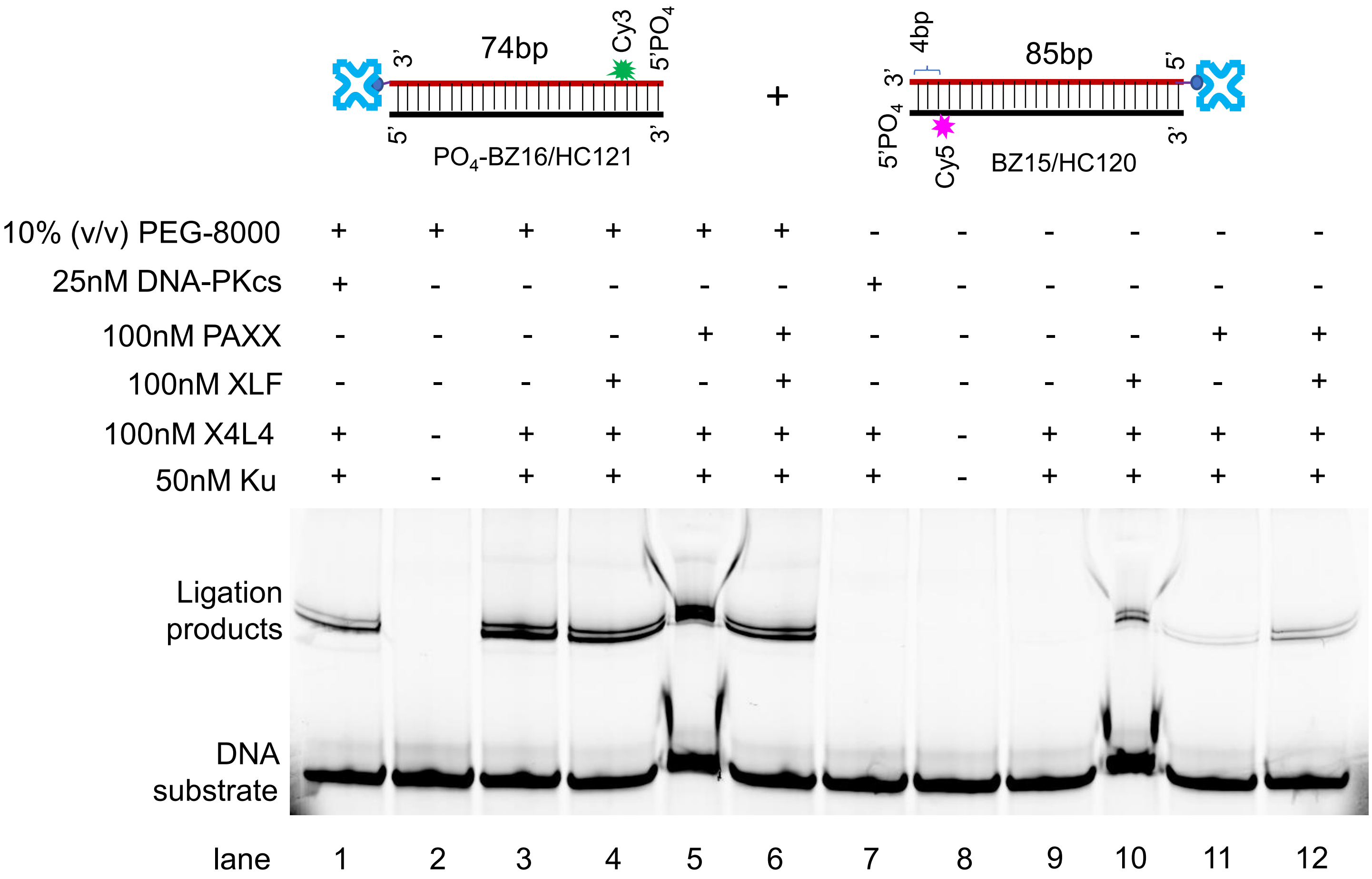

Supplementary Figure 9. Blunt end ligation stimulated by NHEJ components.

Another illustration of blunt end ligation mediated by different combinations of NHEJ components in the presence and absence of 10% (v/v) PEG-8000. The aberrant mobility in lanes 5 and 10 is due to a higher concentration of glycerol used for loading. BZ15 was synthesized with a 5' PO_4.

Source data are provided as a Source Data2 file.

Supplementary Table

Supplementary Table 1. DNA Oligos used in this study

Name	Sequence (5’->3’)	comments
HC116	/5BiosG/CGA TAG TGG GTT CAG CAG GCA TTG TGC TAT GAT CAA CCG AAT CTG TAC ATA TAT CAG TGT CTG CAT CGT CGA CCT TGG AGG CAT C GGGG -3’	
HC120	/5BiosG/CGA TAG TGG GTT CAG CAG GCA TTG TGC TAT GAT CAA CCG AAT CTG TAC ATA TAT CAG TGT CTG CAT CGT CGA CCT TGG AGG CAT C-3’	
HC121	5’-CGT TAA GTA TCT GCA TCT TAC TTG ATG GAG GAT CCT GTC ACG TGC TAG ACT ACT GGT CAA GCG CAT CGA GAA CC-3’	
BZ15	/5Phos/GAT G /iCy5/CC TCC AAG GTC GAC GAT GCA GAC ACT GAT ATA TGT ACA GAT TCG GTT GAT CAT AGC ACA ATG CCT GCT GAA CCC ACT ATC G -3’	5’OH-BZ15: BZ15 was treated with SAP to remove 5’-PO_4
BZ16	5’-GG TT/ iCy3/C TCG ATG CGC TTG ACCA GTA GTC TAG CAC GTG ACA GGA TCC TCC ATC AAG TAA GAT GCA GAT ACT TAA CG /3Bio/	5’PO_4-BZ16: BZ16 was treated with PNK to add 5’-PO_4
BZ18	5’-CGT TAA GTA TCT GCA TCT TAC TTG ATG GAG GAT CCT GTC ACG TGC TAG ACT ACT GGT CAA GCG CAT CGA GAA CC CCCC-3’	
BZ24	5’-GATCC TC CAT CAA GTA AGA TGC AGA TAC TTA ACG TTTTTT CGT TAA GTA TCT GCA TCT TAC TTG ATG GAG-3’	Treated with PNK to add 5’-PO_4
BZ35	/5Phos/GG TT/ iCy3/C TCG ATG CGC TTG ACCA GTA GTC TAG CAC GTG ACA G-3’	5’OH-BZ35: BZ35 was treated with SAP to remove 5’-PO_4
BZ52	5’-GAT CCT GTC ACG TGC TAG ACT ACT GGT CAA GCG CAT CGA GAA CC-3’	Treated with PNK to add 5’-PO_4
BZ69	5’-GAT GCC TCC AAG GTC GAC GAT GCA GAC ACT GAT ATA TGT A /iCy5/ CA GAT TCG GTT GAT CAT AGC ACA ATG CCT GCT GAA CCC ACT ATC G-3’	Mid-point labeled probe
BZ42	5’-Cy5-GGA CTG CCG CCT GGG GAG CCG CAC GAC GAC ACG ACA AAG-Biotin-3’	For FRET standard
BZ43	5’-CGT GTC GTC GTG CGG CTC CCC AGG CG/Cy3/G CAG TCC-3’	For FRET standard
BZ44	5’-CGT GTC GTC GTG CGG CTC CCC AG /Cy3/ G CGG CAG TCC-3’	For FRET standard
BZ45	5’-CGT GTC GTC GTG CGG CTC C /Cy3/ CC AGG CGG CAG TCC-3’	For FRET standard
BZ46	5’-CGT GTC GTC GTG CG /Cy3/ G CTC CCC AGG CGG CAG TCC-3’	For FRET standard
GW21	5’-TTAGGAAAATTTGTTTCATAGT-3’	For DNA-PKcs kinase activity
GW22	5'-ACTATGAAACAAATTTTCCTAA-3'	For DNA-PKcs kinase activity

Supplementary Discussion

Ku and X4L4 are Sufficient for Synapsis and This Does Not Require DNA-PKcs.

Our work here clearly shows that while Ku alone is not sufficient for synapsis of blunt DNA ends, Ku plus X4L4 is sufficient for a level of synapsis that can persist for several seconds to minutes. DNA-PKcs is not essential for and has little effect on the **FS** mediated by Ku and X4L4. The DNA ends used here do not have a 5' PO_4, and so the synapsis we observe is not due to covalent ligation.

A previous study by Graham et al. showed that Ku and DNA-PKcs could mediate the formation of a synaptic complex, in which no FRET signal could be detected between the donor and acceptor dyes located at the ends of two separate duplexes [1]. They inferred that DNA-PKcs kinase activity is not required for this kind of synapsis. Here we found Ku and X4L4 can mediate efficient **FS** and DNA-PKcs is not required for the **FS**. Also, DNA-PKcs does not have a significant effect on the **FS** mediated by Ku and X4L4. Since the synaptic complex of Ku plus DNA-PKcs observed by Graham et al. exhibits no FRET, we cannot exclude the possibility that the two synapsis pathways, either by Ku and X4L4, or by Ku and DNA-PKcs, simultaneously exist in the reaction system. Further experiments using a two-laser system as Graham et al. did might provide more information on this issue. Importantly, the lengths of naked dsDNA in our system are ~80 bp, whereas Graham et al. used dsDNA substrates with 1 kb long. Both systems are informative, but DSBs *in vivo* are unlikely to have a kb of naked DNA exposed.

Here we found the PCA plus PCD oxygen scavenger system severely reduces the kinase activity of DNA-PKcs. The same PCA plus PCD system was used in the study by Graham et al[1]. Though they proposed that DNA-PKcs kinase activity is required for transition from one synaptic state to another, this conclusion must be re-evaluated because the DNA-PKcs kinase was almost certainly inactive due to the use of PCA plus PCD.

Given the requirement of DNA-PKcs for activation of the endonuclease activity of Artemis at DNA ends[2], we speculate that instead of being required for blunt end synapsis, DNA-PKcs plus Artemis may be important for processing incompatible DNA ends prior to synapsis. Although DNA-PKcs does not show any significant effect on end synapsis in our *in vitro* highly purified system here, there is always the possibility that a protein not included

could reveal such a role for DNA-PKcs or that DNA-PKcs *in vivo* is aided by other proteins for end synapsis. Future work is required to address this possibility.

The flexibility and the physical gap between the two dsDNA ends within the **FS** complex may facilitate the end processing by other NHEJ factors, such as nucleases and polymerases [3-5]. Our previous results indicate that L4 can stimulate the nuclease activity of Artemis for end processing [6]. The physical gap between the two ends within **FS** complex might provide enough space for Artemis activity.

Our results show that DNA-PKcs does not have a significant effect on the structure of the **FS** complex formed by Ku and X4L4. Nevertheless, the flexibility of the DNA ends held by Ku and X4L4 in the **FS** may facilitate nucleolytic end processing by DNA-PKcs-activated Artemis. Moreover, the flexibility and proximity for FRET detection of the two dsDNA ends within the **FS** complex may facilitate transient annealing states that represent 'sampling' of the ends for potential microhomology [3,7]. Actually, our previous results suggest that once the ends can transiently pair via microhomology at the ends, Ku and X4L4 are sufficient to promote **CS** complex formation without XLF [8].

The 5' OH at the dsDNA ends precludes ligation and thus permits us to focus only on the synapsis process. In addition to its requirement for covalent ligation, the 5' PO4 at each end could well have some effects on synapsis. Further work will be required to examine this issue.

PAXX Can also Drive Two dsDNA Ends into Close Proximity.

PAXX is known to promote NHEJ in cells [9-11]. Our synapsis results here show that PAXX indeed can drive the dsDNA ends into a close synapsis (**CS**). That PAXX can independently function like XLF to promote **CS** but at a lower efficiency is in agreement with previous findings that PAXX is dispensable for normal mouse development [12-14] and V(D)J recombination *in vivo* [15,16]. It is also in agreement with *in vitro* biochemical studies showing that PAXX and XLF have overlapping functions in ligation [16]. The consistent nature of our smFRET with these *in vivo* results suggest that it is the capability for synapsis that limits the NHEJ activities of different factors. This emphasizes the importance and rate-limiting nature of the synapsis step.

PAXX can interact with Ku70 through its C-terminal end, but with a lower affinity compared to XLF binding to Ku [16], which may explain the much lower efficiency of **CS** complex formation promoted by PAXX. The overlapping function of PAXX and XLF, and the lower **CS** formation efficiency of PAXX suggest that the role of PAXX in synapsis is masked by XLF, as observed in ligation [16].

Synapsis Stimulated by XLF is Primarily End-to-End.

Previous structural investigations and single molecule studies suggested that X4-XLF or X4-XLF-L4 can form filaments, which could be important for these proteins to mediate synapsis [8,17-20]. Therefore, the filament model was proposed to mediate synapsis at the earliest stages of NHEJ. XLF promotes the formation of the **CS** complex, in which the two dsDNA molecules make contact at the ends, which indicates that XLF facilitates end-to-end synapsis. The result that XLF can stimulate the formation of end-to-end **CS** complex, suggests a non-filament role for XLF in promoting synapsis, given that a filament interaction primarily occurs in the mid-section of the dsDNA. Though synapsis could be detected using the duplex with the FRET acceptor labeled at a recessed (mid-point) position in the duplex, the number of detected non-zero FRET molecules is much lower than that found when the acceptor is at the end. This further illustrates that synapsis stimulated by XLF is primarily end-to-end. Importantly, though not likely at play in our purified system, it is quite possible that filaments of XLF could be relevant for chromatinized DNA. Eventual high-resolution cellular work and utilization of chromatinized DNA substrates in biochemical systems will be valuable in this regard.

Supplementary References

1. Graham, T.G., Walter, J.C. & Loparo, J.J. Two-Stage Synapsis of DNA Ends during Non-homologous End Joining. *Mol Cell* **61**, 850-8 (2016).
2. Ma, Y., Pannicke, U., Schwarz, K. & Lieber, M.R. Hairpin Opening and Overhang Processing by an Artemis/DNA-Dependent Protein Kinase Complex in Nonhomologous End Joining and V(D)J Recombination. *Cell* **108**, 781-794 (2002).
3. Daley, J.M., Laan, R.L.V., Suresh, A. & Wilson, T.E. DNA joint dependence of pol X family polymerase action in nonhomologous end joining. *J. Biol. Chem.* **280**, 29030-7 (2005).
4. Daley, J.M., Palmbos, P.L., Wu, D. & Wilson, T.E. Nonhomologous end joining in yeast. *Ann. Rev. Genet.* **39**, 431-451 (2005).
5. Daley, J.M. & Wilson, T.E. Rejoining of DNA double-strand breaks as a function of overhang length. *Mol Cell Biol* **25**, 896-906 (2005).
6. Gerodimos, C.A., Chang, H.H.Y., Watanabe, G. & Lieber, M.R. Effects of DNA end configuration on XRCC4-DNA ligase IV and its stimulation of Artemis activity. *J Biol Chem* **292**, 13914-13924 (2017).
7. Pannunzio, N.R. & Lieber, M.R. Concept of DNA Lesion Longevity and Chromosomal Translocations. *Trends Biochem Sci* **43**, 490-498 (2018).
8. Reid, D.A. et al. Organization and dynamics of the nonhomologous end-joining machinery during DNA double-strand break repair. *Proc Natl Acad Sci U S A* **112**, E2575-84 (2015).
9. Ochi, T. et al. DNA repair. PAXX, a paralog of XRCC4 and XLF, interacts with Ku to promote DNA double-strand break repair. *Science* **347**, 185-188 (2015).
10. Xing, M. et al. Interactome analysis identifies a new paralogue of XRCC4 in non-homologous end joining DNA repair pathway. *Nat Commun* **6**, 6233 (2015).
11. Craxton, A. et al. XLS (c9orf142) is a new component of mammalian DNA double-stranded break repair. *Cell Death and Differentiation* **22**, 890-897 (2015).
12. Gago-Fuentes, R. et al. Normal development of mice lacking PAXX, the paralogue of XRCC4 and XLF. *FEBS Open Bio* **8**, 426-434 (2018).
13. Liu, X., Shao, Z., Jiang, W., Lee, B.J. & Zha, S. PAXX promotes KU accumulation at DNA breaks and is essential for end-joining in XLF-deficient mice. *Nat Commun* **8**, 13816 (2017).
14. Balmus, G. et al. Synthetic lethality between PAXX and XLF in mammalian development. *Genes Dev* **30**, 2152-2157 (2016).
15. Lescale, C. et al. Specific Roles of XRCC4 Paralogs PAXX and XLF during V(D)J Recombination. *Cell Rep* **16**, 2967-79 (2016).
16. Tadi, Satish K. et al. PAXX Is an Accessory c-NHEJ Factor that Associates with Ku70 and Has Overlapping Functions with XLF. *Cell Reports* **17**, 541-555 (2016).
17. Andres, S.N., Modesti, M., Tsai, C.J., Chu, G. & Junop, M.S. Crystal structure of human XLF: a twist in nonhomologous DNA end-joining. *Mol Cell* **28**, 1093-101 (2007).
18. Andres, S.N. et al. A human XRCC4–XLF complex bridges DNA. *Nucleic Acids Research* **40**, 1868-1878 (2012).
19. Hammel, M., Yu, Y.P., Fang, S.J., Lees-Miller, S.P. & Tainer, J.A. XLF Regulates Filament Architecture of the XRCC4.Ligase IV Complex. *Structure* **18**, 1431-1442 (2010).
20. Brouwer, I. et al. Sliding sleeves of XRCC4-XLF bridge DNA and connect fragments of broken DNA. *Nature* **535**, 566-9 (2016).
