# Supplementary material for: The essential elements for the noncovalent association of two DNA ends during NHEJ synapsis
Source: Nat Commun. 2019 Aug 9;10:3588. doi: 10.1038/s41467-019-11507-z (PMC6688983; doi:10.1038/s41467-019-11507-z)
Supplement: Supplementary file 3 — Reporting Summary [file 41467_2019_11507_MOESM3_ESM.pdf]

nature research

Corresponding author(s): Eli Rothenberg and Michael R. Lieber

Last updated by author(s): Jun 30, 2019

Reporting Summary

Nature Research wishes to improve the reproducibility of the work that we publish. This form provides structure for consistency and transparency in reporting. For further information on Nature Research policies, see Authors & Referees and the Editorial Policy Checklist.

Statistics

For all statistical analyses, confirm that the following items are present in the figure legend, table legend, main text, or Methods section.

n/a	Confirmed	
☐	☒	The exact sample size (*n*) for each experimental group/condition, given as a discrete number and unit of measurement
☐	☒	A statement on whether measurements were taken from distinct samples or whether the same sample was measured repeatedly
☐	☒	The statistical test(s) used AND whether they are one- or two-sided<br>*Only common tests should be described solely by name; describe more complex techniques in the Methods section.*
☒	☐	A description of all covariates tested
☐	☒	A description of any assumptions or corrections, such as tests of normality and adjustment for multiple comparisons
☐	☒	A full description of the statistical parameters including central tendency (e.g. means) or other basic estimates (e.g. regression coefficient) AND variation (e.g. standard deviation) or associated estimates of uncertainty (e.g. confidence intervals)
☒	☐	For null hypothesis testing, the test statistic (e.g. *F*, *t*, *r*) with confidence intervals, effect sizes, degrees of freedom and *P* value noted<br>*Give P values as exact values whenever suitable.*
☒	☐	For Bayesian analysis, information on the choice of priors and Markov chain Monte Carlo settings
☒	☐	For hierarchical and complex designs, identification of the appropriate level for tests and full reporting of outcomes
☐	☐	Estimates of effect sizes (e.g. Cohen's *d*, Pearson's *r*), indicating how they were calculated

Our web collection on statistics for biologists contains articles on many of the points above.

Software and code

Policy information about availability of computer code

Data collection	iSMS 2.01, Origin Lab Origin 2019, MATLAB R2018a, GraphPad Prism 7, Microsoft Excel 2018
Data analysis	iSMS 2.01, Origin Lab Origin 2019, MATLAB R2018a, GraphPad Prism 7, Microsoft Excel 2018

For manuscripts utilizing custom algorithms or software that are central to the research but not yet described in published literature, software must be made available to editors/reviewers. We strongly encourage code deposition in a community repository (e.g. GitHub). See the Nature Research guidelines for submitting code & software for further information.

Data

Policy information about availability of data

All manuscripts must include a data availability statement. This statement should provide the following information, where applicable:

- Accession codes, unique identifiers, or web links for publicly available datasets
- A list of figures that have associated raw data
- A description of any restrictions on data availability

Almost all data supporting the conclusions are provided in the files of source data1 and source data2. Other data are available from the corresponding authors upon reasonable request.

Field-specific reporting

Please select the one below that is the best fit for your research. If you are not sure, read the appropriate sections before making your selection.

☒ Life sciences ☐ Behavioural & social sciences ☐ Ecological, evolutionary & environmental sciences

For a reference copy of the document with all sections, see nature.com/documents/nr-reporting-summary-flat.pdf

Life sciences study design

All studies must disclose on these points even when the disclosure is negative.

Sample size	The sample size was reflected by the number of independent repeats. For all the reactions, at least two independent repeats were performed.
Data exclusions	No samples were excluded from the analysis.
Replication	All data shown reflect findings that were reproducible. For all the reactions, at least two independent repeats were performed.
Randomization	No randomization method was used.
Blinding	Investigators were not blinded to group allocation during data collection and analysis because the author who assembled the experiments was the same as the one who did the sm-FRET.

Reporting for specific materials, systems and methods

We require information from authors about some types of materials, experimental systems and methods used in many studies. Here, indicate whether each material, system or method listed is relevant to your study. If you are not sure if a list item applies to your research, read the appropriate section before selecting a response.

Materials & experimental systems

n/a	Involved in the study
☒	☐ Antibodies
☐	☒ Eukaryotic cell lines
☒	☐ Palaeontology
☒	☐ Animals and other organisms
☒	☐ Human research participants
☒	☐ Clinical data

Methods

n/a	Involved in the study
☒	☐ ChIP-seq
☒	☐ Flow cytometry
☒	☐ MRI-based neuroimaging

Eukaryotic cell lines

Policy information about cell lines

Cell line source(s)	High Five insect cell used for protein purification was obtained from Invitrogen (B855-02). HEK293T cell used for XLF purification was obtained from Thermo Fisher Scientific.
Authentication	Cell line authentication was performed by the company that sells Hi5 and 293T, and the cells were used after receipt.
Mycoplasma contamination	Mycoplasma contamination tests were performed by the company that sells Hi5 and 293T, and the cells were used after receipt.
Commonly misidentified lines (See ICLAC register)	No commonly-misidentified lines were used.
