# Supplementary figures and images for: The essential elements for the noncovalent association of two DNA ends during NHEJ synapsis
Source: Nat Commun. 2019 Aug 9;10:3588. doi: 10.1038/s41467-019-11507-z (PMC6688983; doi:10.1038/s41467-019-11507-z)

Fig. 6a

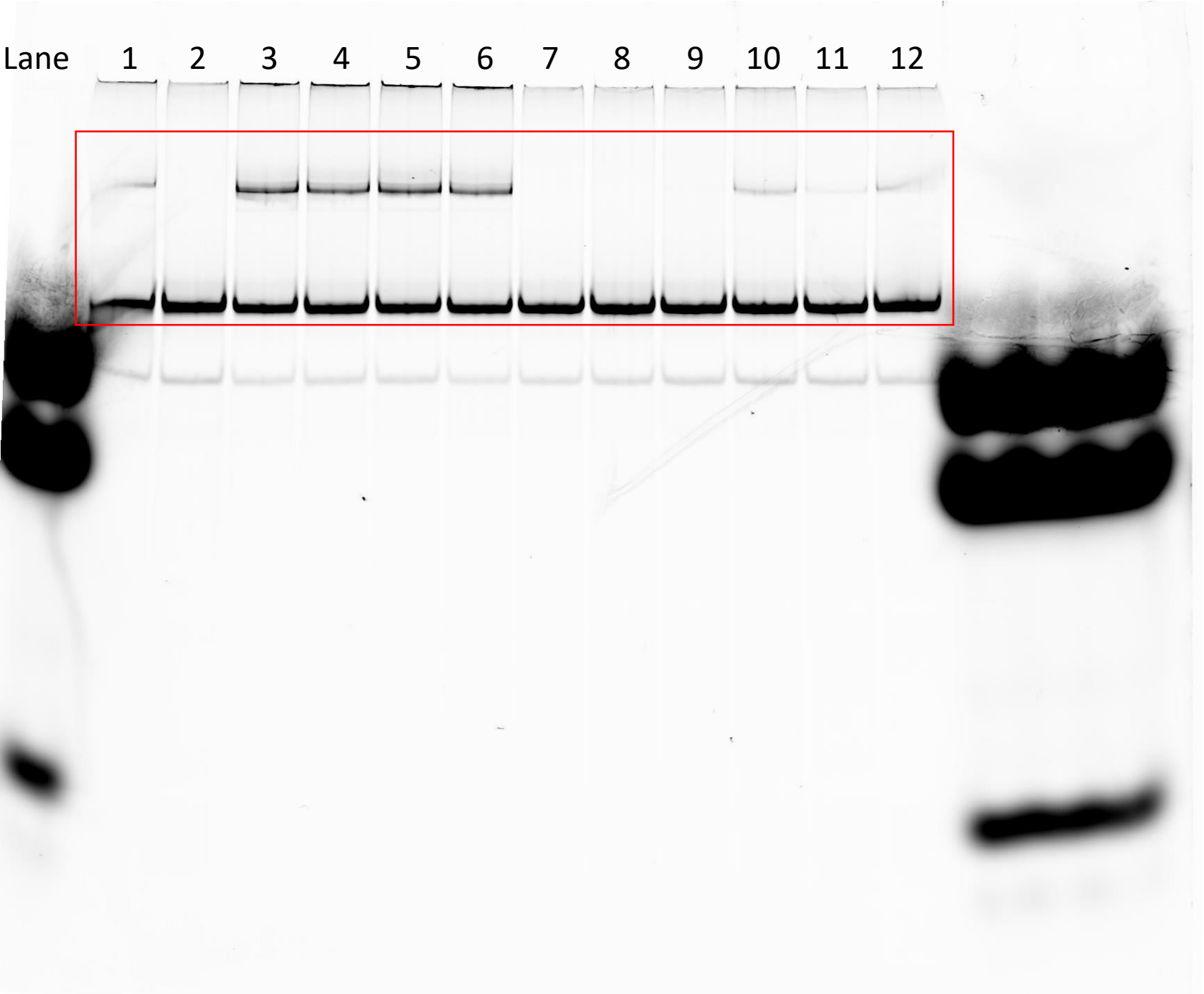

Supplementary Fig. 1b

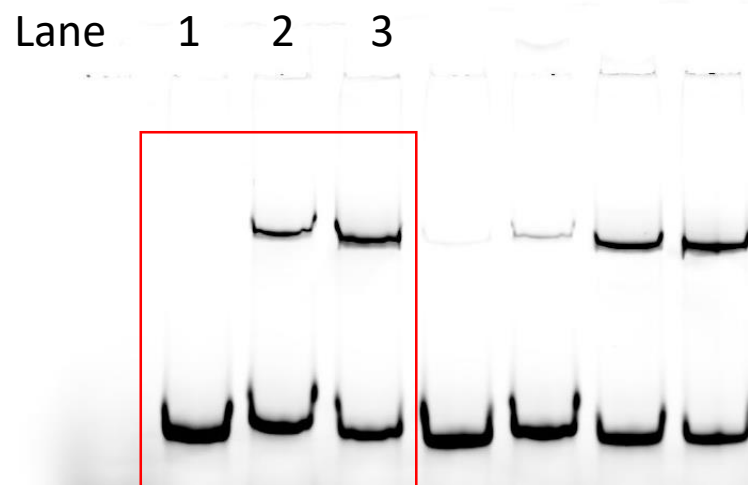

Supplementary Fig. 2

a

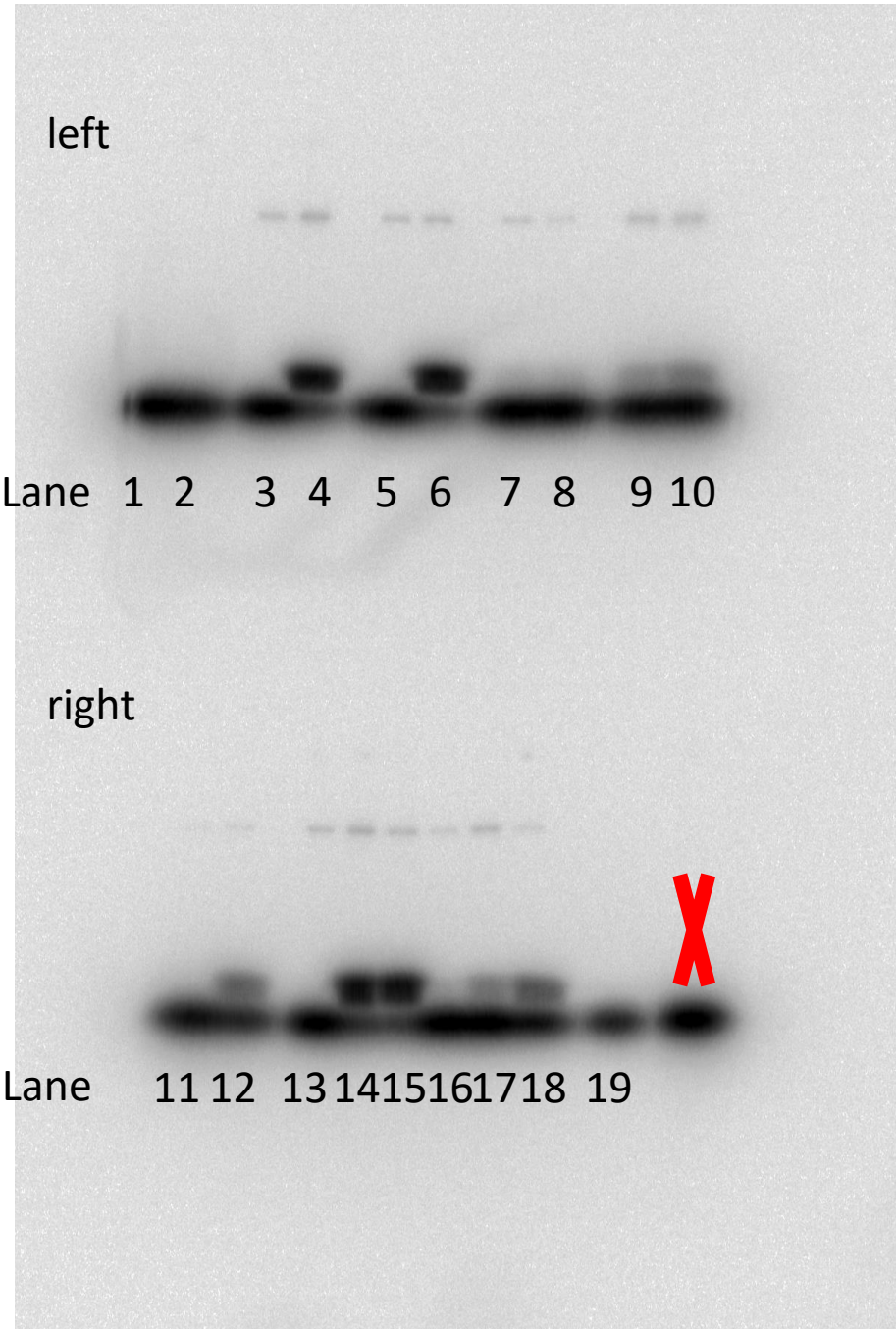

b

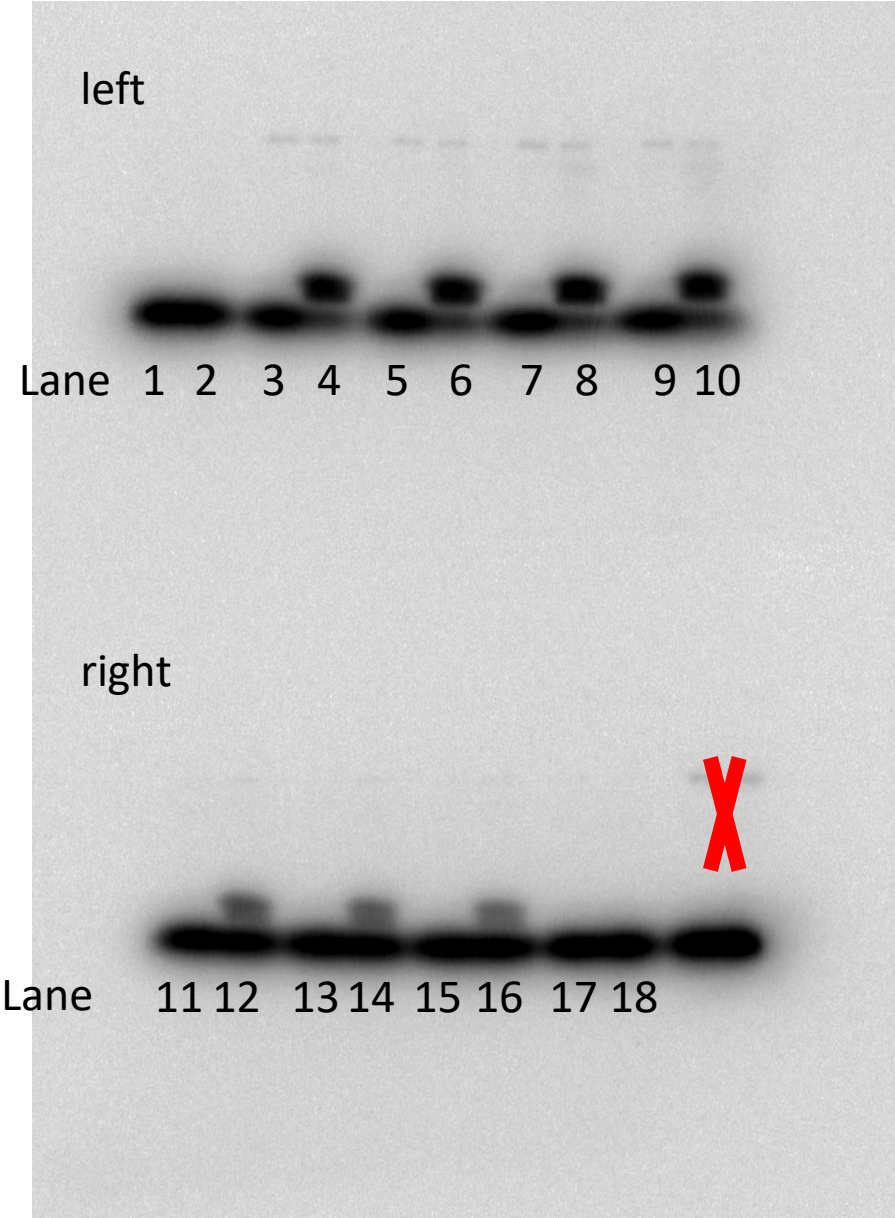

Supplement: Supplementary file 4 — Source Data [file 41467_2019_11507_MOESM4_ESM.zip › Source Data2.pdf]
